# Stereochemistry of 16α-Hydroxyfriedelin and 3-Oxo-16-methylfriedel-16-ene Established by 2D NMR Spectroscopy

**DOI:** 10.3390/molecules14020598

**Published:** 2009-02-04

**Authors:** Lucienir Pains Duarte, Roqueline Rodrigues Silva de Miranda, Salomão Bento Vasconcelos Rodrigues, Grácia Divina de Fátima Silva, Sidney Augusto Vieira Filho, Vagner Fernandes Knupp

**Affiliations:** 1 NEPLAM – Departamento de Química, ICEx, Universidade Federal de Minas Gerais, Av. Antônio Carlos, 6627, Pampulha, CEP 31270-901, Belo Horizonte, Minas Gerais, Brazil; 2 NEProNat - Departamento de Química – FACESA, Universidade Federal dos Vales do Jequitinhonha e Mucuri, Campus II - Rodovia MG 367 – Km 583, nº 5000, CEP 39100-000, Diamantina, Minas Gerais, Brazil; 3 DEFAR, Escola de Farmácia, Universidade Federal de Ouro Preto, Rua Costa Sena, 171, CEP 35400-000, Ouro Preto, Minas Gerais, Brazil; 4 Centro Tecnológico de Minas Gerais, Av. José Cândido da Silveira, 2000, CEP 31170-000, Belo Horizonte, Minas Gerais, Brazil

**Keywords:** *Salacia elliptica*, Celastraceae, 16α-Hydroxyfriedelin, 3-Oxo-16-methylfriedel-16-ene.

## Abstract

Friedelin (**1**), 3β-friedelinol (**2**), 28-hydroxyfriedelin (**3**), 16α-hydroxyfriedelin (**4**), 30-hydroxyfriedelin (**5**) and 16α,28-dihydroxyfriedelin (**6**) were isolated through fractionation of the hexane extract obtained from branches of *Salacia elliptica.* After a week in CDCl_3_ solution, 16α-hydroxyfriedelin (**4**) reacted turning into 3-oxo-16-methylfriedel-16-ene (**7**). This is the first report of a dehydration followed by a Nametkin rearrangement of a pentacyclic triterpene in CDCl_3_ solution occurring in the NMR tube. These seven pentacyclic triterpenes was identified through NMR spectroscopy and the stereochemistry of compound **4** and **7** was established by 2D NMR (NOESY) spectroscopy and mass spectrometry (GC-MS). It is also the first time that all the ^13^C-NMR and 2D NMR spectral data are reported for compounds **4** and **7**.

## Introduction

The genus *Salacia* (Celastraceae) has a great variety of species spread throughout Brazil and other regions of South America [1]. Different bioactive compounds like salacinol [2], kotalonol [2], sesquiterpene alkaloids [3], quinonemethide triterpenes [3] and pentacyclic triterpenes (PCTT) [4] have already been isolated from *Salacia sp.*

**Figure 1:**
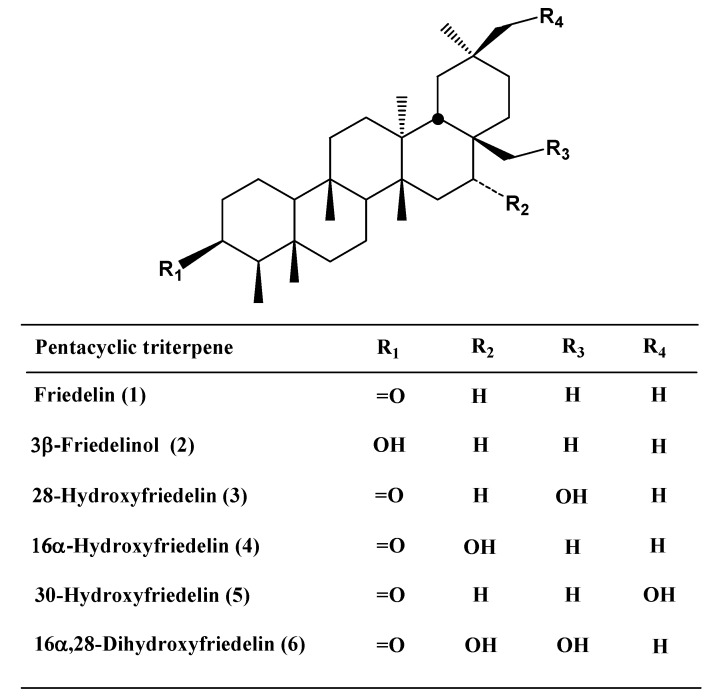
Pentacyclic triterpenes isolated from *Salacia elliptica*.

From the hexane extract of *Salacia elliptica* branches, the following PCTT: friedelin (**1**), 3β-friedelinol (**2**), 28-hydroxyfriedelin (canophyllol, **3**), 16α-hydroxyfriedelin (**4**), 30-hydroxyfriedelin (**5**) and 16α,28-dihydroxyfriedelin (celasdin-B, **6**) (Figure 1) were isolated and identified by TLC comparisons with reference standards and NMR spectroscopy. Compounds **1**, **2**, **3**, **5** and **6** have been isolated from species of the Celastraceae family [5,6,7]. And, this is the first report of the presence of compound **4** in Celastraceae and the isolation of compound **4**, **5** and **6** from specie of the genus *Salacia*.

The triterpene 16α-hydroxyfriedelin (**4**) was previously described as constituent of *Antidesma menasu* Miq.ex.Tul [8]. However, to date, only ^1^H-NMR chemical shifts assignments of **4** have been published. The 2D NMR (HSQC, HMBC, COSY and NOESY) data are essential to elucidate the stereochemistry of PCTTs due to their complex structures [9,10]. The analysis of 2D NMR spectral data contributed to establish the correct chemical shift assignments of all carbons and hydrogens of compound **4**. After the acquisition of 1D NMR data, the CDCl_3_ sample solution was maintained inside the tube for a week, until the 2D NMR experiments were performed. The 2D spectral data obtained showed that compound **4** was not the same. The preliminary analysis indicated that compound **4** had been fully converted to 3-oxo-16-methylfriedel-16-ene (7). This process can be due to a dehydration accompanied by methyl migration of C-17 to C-16, which is in agreement with the Nametkin rearrangement [11,12].

The triterpene **7** had already been produced by the reaction of the C-16-epimer of **4** with MsCl, but, in this case, besides compound **7**, the products 3-oxo-methylfriedel-17(22)-ene and 3-oxo-16-methylfriedel-15-ene were also obtained [13]. 

The literature reports occurrence of olefinic and allylic hydrogen rearrangements in the presence of CDCl_3_, but those reactions were purposely carried out under acidic conditions to study the behavior of compounds [14,15,16]. In the case at hand the transformation of compound **4** into **7** is undoubtedly due to traces of DCl, which is always present in commercial CDCl_3_, unless the solvent is passed through basic alumina (acidity I) immediately before use.

In order to accomplish our initial aim, *i.e.,* establish the complete chemical shifts assignments of **4**, the NMR experiments were repeated, but using pyridine-D_5_ as solvent, and, the results showed that no rearrangement was observed. 

This work describes for the first time the isolation of 16α-hydroxyfriedelin (**4**), 30-hydroxifriedelin (**5**) and 16α,28-dihydroxyfriedelin (celasdin-B, **6**) from *Salacia sp*.; the dehydration of a PCTT (compound **4**) accompanied by structural rearrangement that occurred in CDCl_3_, normally used as a solvent in NMR experiments, and also, the complete 2D NMR spectral data of the compound **4** and **7**. 

## Results and Discussion

The identification of **1**, **2**, **3, 5** and **6** was initially developed through TLC using patterns of PCTT compounds which are commonly isolated from species of the Celastraceae family, and followed by the comparison of the NMR spectral features with literature data [5,17]. The structural elucidation, including the stereochemistry of compounds **4** and **7**, was based on the chemical shifts assignments obtained from 1D (^1^H, ^13^C and DEPT-135) and 2D (HMQC, HMBC, COSY and NOESY) NMR spectral data and mass spectrometry (GC-MS).

The ^1^H-NMR spectrum of **4** presented a double doublet at δ_H_ 4.25 (*J*=7.0 and 10.4 Hz), typical of hydrogen bounded to an oxygenated carbon, suggesting the presence of hydroxyl group in the structure. It also presented a signal at δ_H_ 2.34, characteristic of a hydrogen bonded to a carbon adjacent to a carbonyl group. The ^13^C-NMR of **4** presented a signal at δ_C_ 212.12, attributed to a carbonyl, confirming the presence of a ketone group, and also a signal at δ_C_ 75.70, which was attributed to a carbon bonded to a hydroxyl [17]. The ^13^C-NMR spectral data of **4** was compared to the data of 16β-hydroxyfriedelin [17] since, no ^13^C-NMR data has been reported so far for 16α-hydroxyfriedelin,. This led us to establish the structure of **4** as being 16α-hydroxyfriedelin. 

Compound **4**, dissolved in CDCl_3_, was submitted to 2D NMR experiments aiming to confirm its structure after a week inside the NMR tube. However, a quick analysis of the spectra showed that compound **4** had undergone structural modifications. The signal previously attributed to the H-C-O group hydrogen was no longer observed in the ^1^H-NMR spectrum. In addition, the ^13^C-NMR spectrum showed new signals at δ_C_ 122.57 and δ_C_ 129.50, assignable to olefinic carbons. This modified compound was numbered **7**. Comparison of the spectral data of **4** and **7** suggested that **4** had undergone dehydration, probably due to the residual acidity of CDCl_3_, and acquired a double bound accompanied by methyl migration from C-17 to C-16. This process is the expected outcome of the so-called Nametkin rearrangement [11,12]. To confirm the effect of the acidity of CDCl_3_ in this specific reaction, another 1D NMR experiment was realized with a sample of **4** dissolved in pyridine-D_5_. Similarly to the anterior experiment, the pyridine-D_5_ solution of **4** was also kept inside the NMR tube for a week and then a 2D NMR analysis was carried out. The NMR spectral data obtained using pyridine-D_5_ as solvent did not show any structural modifications of compound **4**. The results suggest that indeed the acidity of CDCl_3_ was sufficient to promote the dehydration of compound **4**. From the HSQC and HMBC spectra of **4** was it possible to correlate each hydrogen signal with its corresponding carbon signal. Through preliminary analysis the chemical shifts assignments of C-16 (δ_C_ 75.70, δ_H_ 4.25) and C-2 (δ_C_ 41.90, δ_H_ 2.34 and δ_H_ 2.44) were identified.

**Figure 2:**
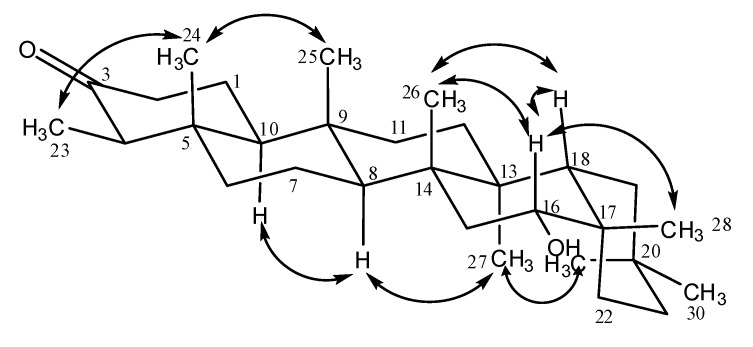
Some correlations observed in the NOESY spectrum of 16α-hydroxyfriedelin (**4**).

In the HMBC spectra correlations of the signal at δ_C_ 212.12 (C-3) with the signals at δ_H_ 2.34, 2.44 (H-2), 1.62, 1.86 (H-1), δ_H_ 0.95 (H-23) and at δ_H_ 2.20 (H-4) were observed. This last one presented correlations with the signals at δ_C_ 7.55 (C-23), 15.08 (C-24), 42.37 (C-5) and at δ_C_ 59.77 (C-10). This last signal correlated with δ_H_ 2.34, 2.44 (H-2), 2.20 (H-4) and δ_H_ 1.51 (H-8). The signal of C-8 (δ_C_ 50.82) presented correlation with the signals at δ_H_ 0.84 (H-25), 0.93 (H-26), 1.32 (H-11), 1.66 and δ_H_ 2.08 (H-15). The signal at δ_H_ 4.25 (H-16) correlated with the signals at δ_C_ 27.76 (C-22), 31.03 (C-28), 37.75 (C-17), 40.25 (C-14 and C-15) and δ_C_ 46.66 (C-18). This signal was correlated to the signals at δ_H_ 1.35 (H-19), 1.34 (H-27) and δ_H_ 1.37 (H-28). The signal of H-28 correlated with the δ_C_ 27.76 (C-22) and δ_C_ 37.75 (C-17). The signals at δ_H_ 1.05 and δ_H_ 1.09 presented correlations with the signal at δ_C_ 28.62 (C-20), 34.28 (C-19) and δ_C_ 34.97 (C-21). These two signals could only be attributed to H-29 and H-30. Both signals have close chemical shifts values and, for this reason, it becomes difficult to distinguish these two methyl groups through the HMBC spectrum. However, they could be distinguished and the stereochemistry of **4** established from the NOESY spectrum, since it was possible to observe NOEs between H-16 axial and H-15 equatorial, H-18, H-26 and H-28. NOEs were also observed between H-23 and H-24; and between H-24 and H-25. This last signal presented NOE with the signal of H-26 and this one correlated with H-18. It was observed correlation between H-27 and H-29 which presented correlation with H-21 equatorial. The signal of H-27 was correlated with the signal of H-8, which presented correlation with the signal of H-10. Some correlations, observed in the NOESY spectrum of compound **4,** are shown in Figure 2. 

The 1D NMR spectral data of 16β-hydroxyfriedelin [17] are compared with those of compound **4** and the complete 1D/2D NMR data for compounds **4** and **7** are presented in Table 1. 

**Table 1:** ^1^H (400 MHz) and ^13^C (100 MHz) NMR spectral data of 16α-hydroxyfriedelin (**4**) (δ values, Py-d_5_) and 3-oxo-16-methylfriedel-16-ene (**7**) (δ values, CDCl_3_) * (Literature data of 16 -hydroxyfriedelin) [17].

No	*δ*_C_ (lit) *	*δ*_C_ (4)	*δ*_H_ (4)	HMBC	*δ*_C_ (7)	*δ*_H_ (7)	HMBC
1	22.3	22.77	1.62 ax 1.86 eq		22.32	1.68 ax 1.98 eq	
2	41.6	41.90	2.34 eq 2.44 ax		41.55	2.39 ax 2.45 eq	
3	212.5	212.12	-	1, 2, 4, 23	213.21	-	4, 23
4	58.3	58.28	2.20, m	23, 24, 5, 10	58.25	2.27, m	
5	42.3	42.37	-		42.07	-	4, 10
6	41.4	41.54	1.54, m		41.09	1.23 eq 1.74 ax	
7	18.6	18.95	1.64, m		18.23	1.39 eq 1.48 ax	
8	53.5	50.82	1.51, m	12, 15, 25, 26	50.18	1.38, m	6, 10, 11, 25, 26
9	37.6	37.97	-		37.53	-	
10	59.7	59.77	1.56, m	2, 4, 8	59.25	1.55, m	8
11	35.8	35.64	1.32, m		35.50	1.27 ax 1.51 eq	
12	30.8	30.42	1.29 ax 1.40 eq		28.14	1.35, m	
13	39.3	39.95	-		37.29	-	8, 12, 26, 27
14	40.1	40.25	-		37.65	-	
15	44.4	40.25	1.66 ax, m 2.08 eq, m		43.07	1.52 eq 1.58 ax	
16	75.6	75.70	4.25 *J* = 7.0; 10.4 Hz	14, 15, 17, 18, 22, 28	122.57	-	28
17	32.1	37.75	-		129.50	-	28
18	44.8	46.66	1.63, m	19, 27, 28	40.43	1.87, m	12, 19
19	35.8	34.28	1.35, m		37.69	1.03 ax 1.29 eq	
20	28.0	28.62	-		30.00	-	
21	32.1	34.97	1.62, m		38.33	1.19 eq 1.36 ax	
22	36.0	27.76	1.97, m 2.05, m		24.60	1.91 ax 2.48 eq	
23	6.8	7.55	0.95, d *J* = 6.7 Hz	3, 4, 5	6.84	0.88, d *J* = 6.8	3, 4, 5
24	14.7	15.08	0.69, s	4, 5, 10	14.64	0.72, s	
25	18.2	19.43	0.84, s	8, 9, 10	17.20	0.86, s	
26	20.1	17.70	0.93, s	8, 13 14 15	16.43	0.75, s	8, 13, 14
27	21.5	20.02	1.34, s	12, 13, 14, 18	16.59	0.84, s	13, 14, 18
28	24.9	31.03	1.37, s	17, 22	19.62	1.59, s	15, 16, 17
29	30.8	32.67	1.09, s	19, 20, 21	33.07	0.92, s	19, 20, 21
30	35.5	36.95	1.05, s	19, 20, 21	24.45	0.94, s	19, 20, 21

The ^1^H-NMR spectrum of **7** showed multiple signals in the region between δ_H_ 0.70 and δ_H_ 2.50. As mentioned, a lack of signals at the region of the H-C-O hydrogen was observed. The ^13^C-NMR spectrum presented a signal at δ_C_ 213.21, which was assigned to a carbonyl group, and two non-hydrogenated carbon signals at δ_C_ 122.57 and δ_C_ 129.50 that were attributed to olefinic carbons. All chemical shifts of hydrogens and carbons of compound 7 were assigned through the HMBC spectra. The signal at δ_C_ 213.21 (C-3) correlated with the signals at δ_H_ 2.27 (H-4) and δ_H_ 0.88 (H-23). This last one showed correlation with the signal at δ_C_ 42.07 (C-5), which presented correlation with δ_H_ 2.27 (H-4) and δ_H_ 1.55 (H-10). This last one correlated with the signal at δ_C_ 50.18 (C-8). The signal of C-8 correlated with the signal at δ_H_ 1.74 (H-6), 1.55 (H-10), 1.51 and δ_H_ 1.27 (H-11), and also with the two methyl signals at δ_H_ 0.75 and 0.86, attributed to H-26 and H-25. The signal at δ_H_ 0.75 presented correlation with the signal of C-13 (δ_C_ 37.29), then this signal could only be associated to H-26, and consequently, the signal at δ_H_ 0.86 was attributed to H-25. The signal of C-13 (δ_C_ 37.29) correlated with δ_H_ 1.38 (H-8), 1.35 (H-12) and δ_H_ 0.84 (H-27) and this last one was also correlated with δ_C_ 40.43 (C-18). The signal of C-18 correlated with the signals at δ_H_ 1.35 (H-12), 1.03 and δ_H_ 1.29 (H-19). Also were observed correlations between the signals of olefinic carbon at δ_C_ 122.57 (C-16) and δ_C_ 129.50 (C-17) with the signal of methyl hydrogen at δ_H_ 1.59 attributed to H-28, because it is the only methyl group able to correlate with carbons C-16 and C-17. The signal of H-28 presented yet a correlation with the signal at δ_C_ 43.07 (C-15). 

**Figure 3:**
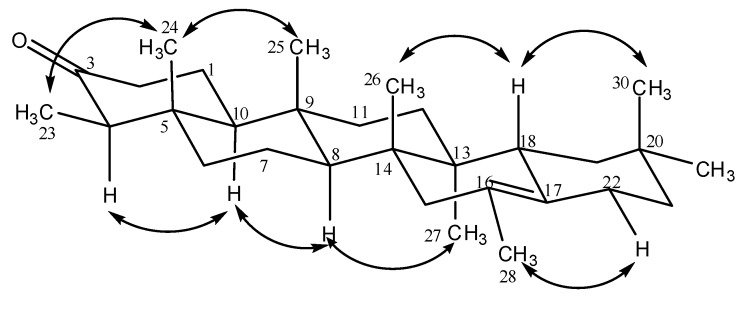
Some correlations observed in NOESY spectrum of 3-oxo-16-methylfriedel-16-ene (**7**).

The analysis of the NOESY spectrum permitted us to determine the stereochemistry of compound **7**. It was possible to observe NOEs between H-30, H-18 and H-19 equatorial. These correlations indicated that the E ring has a chair conformation. NOEs were also observed between H-18 and H-26, between H-25, H-24 and H-26, and also between H-27 and H-11 axial, H-19 axial, H-12 equatorial, H-15 axial and H-8. It was possible to observe correlations between H-23/H-24 and H-10/H-4, and this last one presented correlation with H-2 axial. The H-28 hydrogen presented a NOE with H-22 equatorial. Some correlations observed in the NOESY spectrum of compound **7** are shown in Figure 3. The complete 1D/2D NMR spectral data of 3-oxo-16-methylfriedel-16-ene (**7**) are presented in Table 1. The mass spectrum of **4** did not show the molecular ion at m/z 442, but rather showed peaks at m/z 411 (M-OH,-CH_2_) and m/z 273, confirming it to be a friedelane derivative [18]. On the other hand, the mass spectrum of compound **7** showed the molecular ion at m/z 424, corroborating the molecular formula C_30_H_48_O.

By the data obtained through NMR and CG/MS was possible to confirm that the structure of PCTT **4** was modified by a process of dehydration accompanied by methyl rearrangement induced by the acidity CDCl_3_ that is normally used as NMR solvent, producing compound **7**. To the best of our knowledge, this is the first report of the Nemetkin rearrangement of a pentacyclic triterpene dissolved in CDCl_3_, inside an NMR tube, and also the isolation of compounds **1** to **6** from *Salacia elliptica*.

## Experimental

### General

Melting points (uncorrected) were determined on a Mettler FP 80 HT. The IR spectra were obtained on a Perkin Elmer, Spectrum One (SN 74759) spectrophotometer. Plates of silica gel G-60 were previously activated at 100°C/30min, and developed with an acidic soln. of vanillin in perchloric acid [19] after the TLC processes. Column chromatography (CC) processes were developed using silica gel (Merck, 230-400 mesh). GC-MS analysis was carried out on a Hewlett Packard HP5890 instrument, equipped with a HP 7673 injector, HP-1 (50 m x 0.25 mm i.d. x 0.2 mm film) column and helium as mobile phase. Operating conditions: injector temperature at 300 °C, 2 μL of sample solution (10 μmg/100 μL); splitless of 30s followed by split 1:40 (30 psi stream pressure). The initial oven temperature was 200 ºC/3min, followed by 10°C/min until 300ºC and with 40 min holding time. Interface: quadrupole mass spectrometer model HP 5971, electron impact ionization, 70 eV potential. 

### NMR spectra

NMR spectra were recorded on a Bruker DRX400-AVANCE spectrometer operating at 400 and 100 MHz at 27 ^0^C equipped with a direct detection 5 mm ^1^H/^13^C dual probe and a 5 mm inverse probe with z-gradient coil. The solvent was CDCl_3_ or pyridine-D_5_. Compound **4** (about 10 mg) was dissolved in 0.7 mL of CDCl_3_ or pyridine-D_5_, and transferred to a 5 mm o.d. tube. The chemical shifts are reported in ppm using TMS (0 ppm) as internal standard. One-dimensional ^1^H- and ^13^C-NMR spectra were acquired under standard conditions. Two-dimensional inverse hydrogen-detected heteronuclear shift correlation spectra were obtained by HSQC pulse sequence [1J(C, H)] and HMBC pulse sequence [^n^J (C, H), n = 2 and 3], ^1^H homonuclear correlation spectroscopy (COSY) and homonuclear 2D-NOESY (mixing time = 441 ms) experiment were used to confirm the assignments of all carbons and hydrogens of the compounds.

### Plant Material and Compound Isolation

Leaves and branches of *Salacia elliptica* were collected in the Mata Samuel de Paula, Nova Lima region, Minas Gerais, Brazil, in August of 2005. They were separated, dried at room temperature (r.t.) and powdered in a mill. The branches (1158 g) were successively submitted to exhaustive extraction at r.t. with solvents of different polarity. Each solvent was removed under vacuum furnishing the hexane (6.11 g), ethyl acetate (8.16 g) and finally ethanol (144.5 g) extracts. 

During the hexane removal, the formation of a white solid was observed. The solid material (0.98 g) was separated by filtration. This material was fractionated by silica gel CC eluted with dichloromethane, ethyl acetate and ethanol pure or in mixture of gradient polarity, obtaining 69 fractions. Fraction 4-5 was analyzed by TLC and GC together with PCTT standards and identified as friedelin (**1**). By these comparative analyses also was possible to identify fraction 6-7 as a mixture of **1** and 3β-friedelinol (**2**). Fraction 10-15 gave a white solid material (mp. 279.5-281.8 °C). Its ^1^H- and ^13^C-NMR data were compared with the literature data [17] and identified as 28-hydroxyfriedelin (canophyllol, **3**). Fraction 18 (200 mg) was submitted to TLC, which showed the presence of only three components. It was then separated by CC using CHCl_3_, ethyl acetate and ethanol as eluents furnishing 16α-dihydroxyfriedelin (**4**, 18 mg, m.p. 238.7-244.9 ^o^C) and compound **5** (102.5 mg, m.p. 270.6-278.7 °C), identified as 30-hydroxyfriedelin. After solvent evaporation, fraction 46-47 presented as a white solid (20.2 mg, m.p. 170-172.5 ^0^C). By comparison of its NMR spectral data with the literature [5] this solid material was identified as 16α,28-dihydroxyfriedelin (**6**). For the NMR analysis, a sample of **4** (10 mg) was dissolved in CDCl_3_ (0.7 mL) and placed within the NMR tube. After the acquisition data of 1D NMR, the solution was kept inside the tube during a week, and then submitted to 2D NMR experiments. After CDCl_3_ evaporation the solid was recovered and named as **7** (melting point 240-242 ^o^C). 
